# Splicing Variants of *SERPINA1* Gene in Ovine Milk: Characterization of cDNA and Identification of Polymorphisms

**DOI:** 10.1371/journal.pone.0073020

**Published:** 2013-08-23

**Authors:** Cinzia Marchitelli, Alessandra Crisà, Elisa Mostarda, Francesco Napolitano, Bianca Moioli

**Affiliations:** Consiglio per la Ricerca e la sperimentazione in Agricoltura - CRA, PCM, Animal Production Research Centre, Monterotondo, Italy; University of Sydney, Australia

## Abstract

The serine protease inhibitor, clade A, member 1 (*SERPINA1*) is the gene for a protein called alpha-1-antitrypsin (AAT), which is a member of the serine protease inhibitor (serpin) superfamily of proteins. By conformational change, serpins control several chemical reactions inhibiting the activity of proteases. AAT is the most abundant endogenous serpin in blood circulation and it is present in relatively high concentration in human milk as well as in bovine and porcine colostrum. Here we report for the first time the molecular characterization and sequence variability of the ovine *SERPINA1* cDNA and gene. cDNAs from mammary gland and from milk were PCR amplified, and three different transcripts (1437, 1166 and 521bp) of the *SERPINA1* gene were identified. We amplified and sequenced different regions of the gene (5’ UTR, from exon 2 to exon 5 and 3’ UTR), and we found that the exon-intron structure of the gene is similar to that of human and bovine. We detected a total of 97 SNPs in cDNAs and gene sequences from 10 sheep of three different breeds. In adult sheep tissues a *SERPINA1* gene expression analysis indicated a differential expression of the three different transcripts. The finding reported in this paper will aid further studies on possible involvement of the *SERPINA1* gene in different physiological states and its possible association with production traits.

## Introduction

Serine protease inhibitor (serpin) superfamily constitutes the largest class of serine/cysteine peptidase inhibitors, currently having >3000 members within Eukarya, Bacteria, Archea and certain viruses [1,2]. These protease inhibitors are involved in many critical biological processes like blood coagulation, fibrinolysis, programmed cell death, development and inflammation [3]. Eukaryotic serpins have been divided into 16 clades [1,4]. There is a high rate of conservation in the structure among the members of serpin family. The average size of protein is 350-400 amino acids (aa) with a molecular weight of 40-50 kDa [3]. The serpin fold is comprised of 3 β sheets (A, B,C) and 7-9 α helices. The regions important for protease inhibition are centered on β sheet A and a stretch of amino acids termed Reactive Center Loop (RCL). The RCL participates in the initial interaction with the target protease, which recognizes it as a substrate and cleaves between two residues termed P1 (N-terminal of the cleavage event) and P1’ (C-terminal of the cleavage event.) The residues on the amino-terminal side of the cleavage are termed P2, P3, and so on, and those carboxi-terminal are termed P2’, P3’ and so on [5].

The interaction of the serpin with the active site of its target protease triggers conformational changes and results in an irreversible serpine-protease complex (named suicide substrate-like inhibitory mechanism) [6].

Alpha-1-antitrypsin (AAT) is a 394-aa, 52 kDa glycoprotein synthesized primarily by hepatocytes, with smaller amounts synthesized by intestinal epithelial cells, neutrophils, pulmonary alveolar cells and macrophages [7,8]. AAT is the most abundant, endogenous serine protease inhibitor in blood circulation and it has been implicated in regulating vital fluid phase biological events such as blood coagulation, fibrinolysis, complement activation, apoptosis, reproduction, tumor progression and inflammatory response [9,10,11]. The primary function of AAT is thought to be the inactivation of neutrophil elastase and other endogenous serine proteases [7,12].

AAT is also present in human milk (range from 0.1 to 0.4 g/l in early lactation, with a subsequent decrease as lactation progresses) and in bovine, porcine and ovine milk [13,14]. A few hypotheses have been suggested regarding the role of protease inhibitors for both the mother and infant [15]. It was postulated that milk AAT might inactivate some of endogenous proteases and protect the infant liver; another possibility is that protease inhibitors affect local proteolytic activity within the mammary gland during colostrum formation. An additional possible role of milk protease inhibitors could be to increase the survival of other milk proteins via partial inhibition of pancreatic proteases, which would influence infant development [15]. Association of polymorphisms of the AAT gene with milk production traits in dairy cattle was demonstrated [16,17,18].

In sheep, Signorelli et al. [19] demonstrated the differential expression of AAT at different lactation stages, comparing the expression of proteins extracted from mammary gland samples of two breeds (Sarda and Gentile di Puglia) dramatically differing in milk traits.

Ten milk samples from ewes of three different breeds (4 Sarda, 3 Gentile di Puglia and 3 Comisana) and eleven different ovine tissue samples (spleen, *semitendinosus* and *longissimus dorsi* muscles, mammary gland, brain, cerebellum, rumen, bladder, adrenal, uterus, liver) from two sheep, Sarda and Gentile di Puglia breed were collected.

cDNAs were synthesized from total RNA extracted from milk and tissue samples. Three different transcripts were cloned and sequenced. SNPs were detected by sequencing and alignment of the longer transcript variant obtained from 10 sheep (3 Comisana, 4 Sarda and 3 Gentile di Puglia).

PCR amplification and sequencing of the *SERPINA1* gene were performed and the whole gene was sequenced in 10 sheep to detect SNPs.

To evaluate a potential impact of the 97 detected SNPs on splicing of *SERPINA1* gene, the Human Splicing Finder (HSF) software [20] was used. To determine the potential deleterious effect of the amino acid changes on protein function we used the Sorting Intolerant From Tolerant (SIFT) software [21].

Based on the aforementioned studies, it was decided to investigate the serine protease inhibitor, clade A, member 1 (*SERPINA1*) gene expression in milk and mammary gland and to elucidate the ovine gene structure. In this manuscript we report the molecular characterization and tissue expression of ovine *SERPINA1* cDNA. Moreover, sequence variability of cDNA and gene in different sheep breeds is described.

## Results

### Identification of the ovine *SERPINA1* cDNA

**Figure 1:**
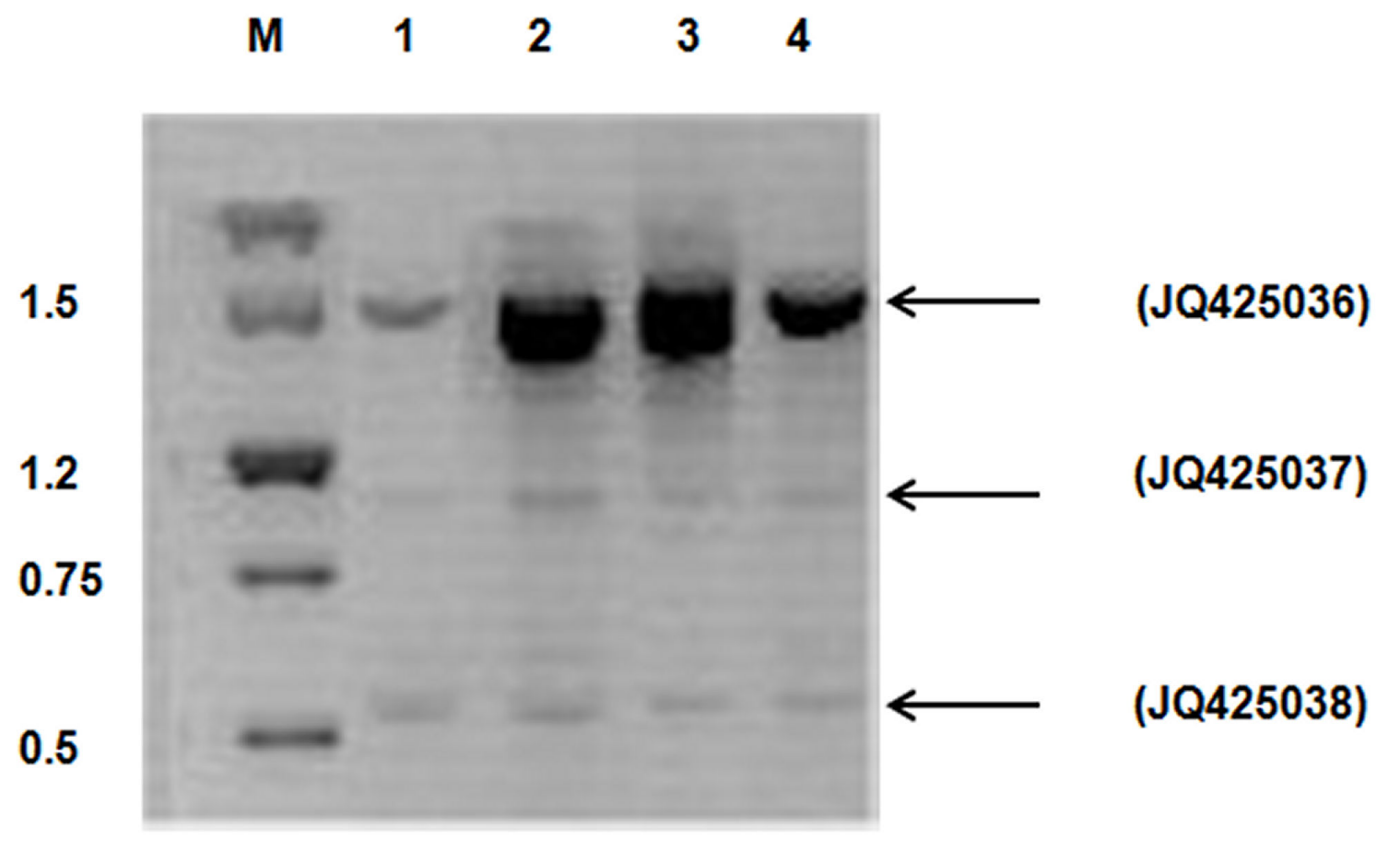
Gel electrophoresis of ovine *SERPINA1* cDNA transcript variants. Molecular weight markers on the left (lane M), cDNA from mammary gland Sarda (lane 1) and Gentile di Puglia (lane 2) breeds, and cDNA from milk cells of Sarda (lane 3) and Gentile di Puglia (lane 4) breeds. The three identified splicing variants are indicated by arrows on right side of pictograph.

Using the primer pair for full length ovine *SERPINA1* cDNA (Table 1, cDNA FWD and cDNA REV) three different transcripts were obtained by PCR using RNA extracted from milk and mammary gland samples (Figure 1). All transcripts showed an untranslated first exon similar to *B. taurus* (NCBI GENE ID280699) and *H. sapiens* (NCBI GENE ID 5265).

**Table 1:** List of primers used to amplify and to sequence the ovine *SERPINA1* cDNA and gene.

**Primer name**	**Sequence 5’->3’**	**Region**
cDNA FWD	CAGAAGCTCCTTCCTCCTGC	I exon
cDNA REV	TTTAATGCCATGGAGGGAAGA	V exon
5’UTR FWD	TGCAGAGCCCTGGGTAAGA	5’UTR
5’UTR REV	CCGTATTTAAGCACTGGACCC	5’UTR
5’UTR FWD Int	AAAGCTTGGTGAGCAGGTGT	5’UTR
5’UTR REV Int	CCGTGCACCACAACTAGAGT	5’UTR
II intron FWD	GCTGGGGTTCTCCAAGGAC	II exon
II intron REV	GTTTGCTCATTCACGTGGAAGTC	III exon
III intron FWD	GACTTCCACGTGAATGAGCAAAC	III exon
III intron REV	CCAGTCACCCAGGACAGTTTTC	IV exon
IV intron FWD	GAAAACTGTCCTGGGTGAACTGG	IV exon
3’UTR FWD	TCTTCCCTCCTCCATGGCATTAAA	V exon
3’UTR REV	TCCAAGAGATAGTGAAGGACAGG	3’UTR
3’UTR FWD Int	TGTGGTCTCTGGCTGGAAAC	3’UTR
3’UTR REV Int	TGGGTAATAATCGTGTCATTAATGG	3’UTR

Primers cDNA FWD, 5’ UTR FWD, 5’ UTR REV and 3’ UTR REV were designed using the bovine transcript ENSBTAT00000045193. Primers II intron FWD, II intron REV, III intron FWD, III intron REV, IV intron FWD, IV intron REV, 3’ UTR FWD were designed using the ovine mRNA sequence (NM_001009799).

The long transcript with an expected length of 1437 bp, revealed the presence of five exons corresponding to an open reading frame (ORF) of 1251 bp (from base 122, exon 2, to base 1372, exon 5) and encoding a putative AAT protein of 416 aa. This protein shows a signal peptide of 24 aa and a RCL of 25 aa in the C-terminal side, like other proteins of the serpin superfamily.

The medium transcript was 1166 bp, displayed a deletion of 271 bp and lacked exon 3. The skip of this exon caused a reading frame shift that resulted in a premature stop codon and generated a protein of 230 aa. This protein was missing regions important for AAT structure and function.

The short transcript was 522 bp, displayed a deletion of 915 bp and lacked exon 2 and 3. The produced protein of 112 aa included only the 25 aa of the RCL motif and the C-terminal.

Our newly sequenced data of *SERPINA1* cDNA transcript variants can be accessed through the following NCBI GenBank accession numbers: transcript variant 1=JQ425036, transcript variant 2=JQ425037 and transcript variant 3=JQ425038.

### Tissue distribution of ovine *SERPINA1* transcripts

**Figure 2:**
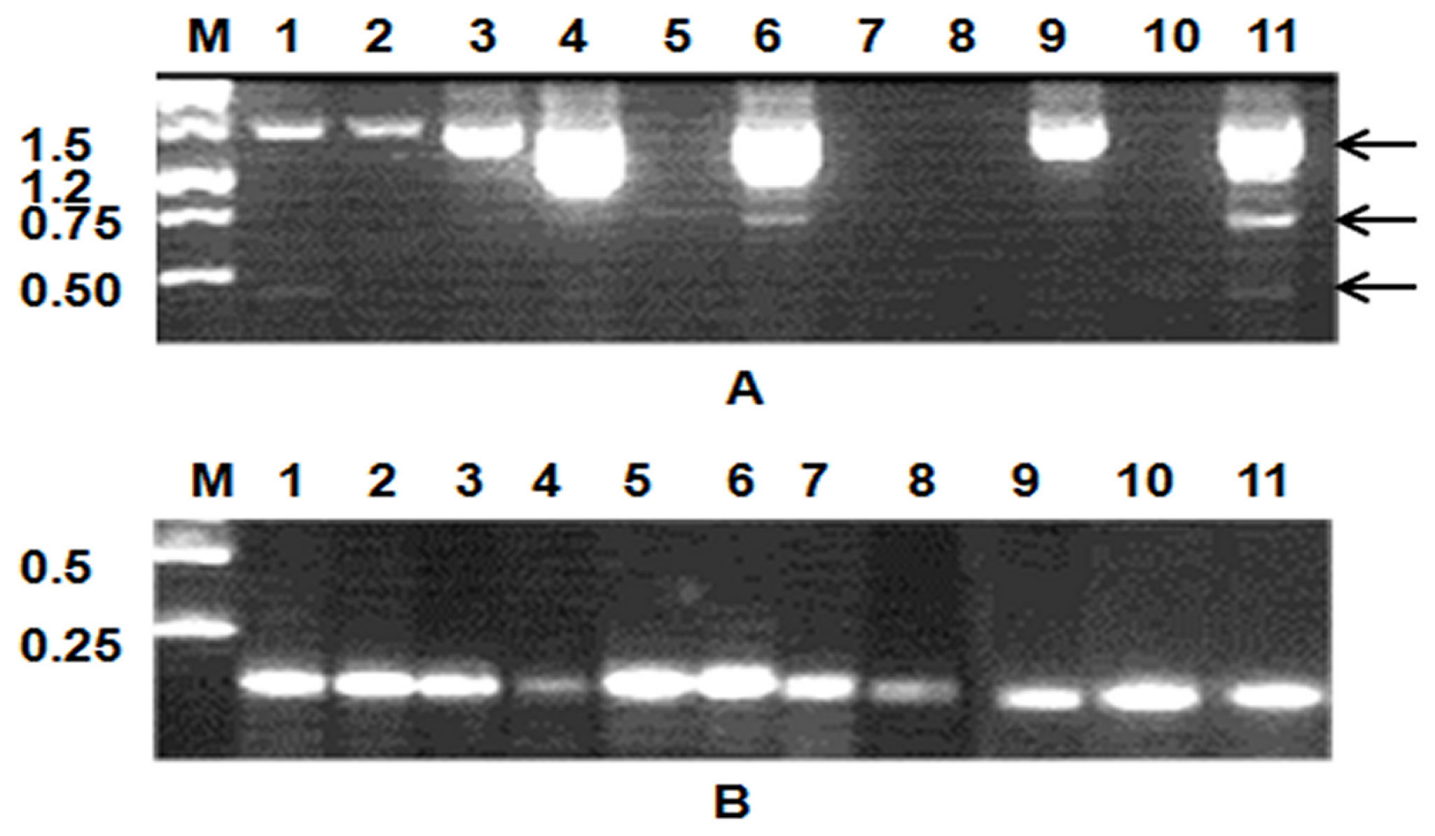
Representative results of *SERPINA1* gene expression in different ovine tissues. A) Expression of *SERPINA1* transcript variants (indicated by arrows on right) and B) expression of ATPB5 control gene in various tissues. Lanes represent molecular weight marker (M) spleen (1), *semitendinosus* muscle (2), longissimus dorsi muscle (3), mammary gland (4), brain (5), cerebellum (6), rumen (7), bladder (8), adrenal (9), uterus (10) and liver (11)..

Tissue distribution of ovine *SERPINA1* transcripts was obtained by RT-PCR of RNA extracted from eleven tissues by using the primer pairs for full length ovine *SERPINA1* cDNA (Table 1). *SERPINA1* gene was differentially expressed among tissues and each tissue displayed a specific profile (Figure 2). Transcripts were completely absent in the rumen, in the bladder and in the uterus; while in other tissues one, two or all three splicing variants were present. *Longissimusdorsi* muscle, mammary gland, cerebellum, adrenal and liver showed a higher expression of the longer transcript than spleen and *semitendinosus* muscle. The intermediate transcript was weakly expressed in mammary gland, brain, cerebellum and adrenal while it was highly expressed in liver. Only spleen, mammary gland and liver showed a weak expression of the short transcript.

### Genomic organization of ovine *SERPINA1* gene

The amplification of the complete *SERPINA1* gene with cDNA FWD and cDNA REV primers (Table 1) showed a product of about 9.0 kbp length as expected in comparison to bovine gene (data not shown). To obtain the sequence of this long amplicon we decided to amplify four PCR products corresponding to Ex1-Int1-Ex2, Ex2-Int2-Ex3, Ex3-Int3-Ex4, Ex4-Int4-Ex5 regions. Also the 5’ UTR and 3’ UTR regions were amplified. The five amplicons corresponding to 5’ UTR, Ex 2-Int2-Ex3, Ex3-Int3-Ex4, Ex4-Int4-Ex5 and 3’ UTR region were 2009 bp, 1495 bp, 1278 bp, 1132 bp and 2082 bp respectively. To date we have not got the intron 1 complete sequence due to the fragment length (Ex1-Int1-Ex2 about 5.0 kbp).

As human and bovine gene, ovine *SERPINA1* was organized into five exons and four introns; the first exon (117 bp) is transcribed but not translated. The other four exons were 643 bp, 271 bp, 148 bp, 193 bp respectively and were separated by three introns of 858 bp, 977 bp, 778 bp respectively; the second exon contained the putative ATG start codon. All the intron-exon boundaries conform to the GT-AG rule [22]. Our new sequence data of *SERPINA1* gene can be accessed through the following NCBI GenBank accession number: JQ436920.

### SNP identification in ovine *SERPINA1* cDNA and gene

We amplified and sequenced the *SERPINA1* long cDNA transcript and five amplicons of the gene of 10 ovine milk samples from multiple breeds (3 Comisana, 4 Sarda and 3 Gentile di Puglia). The alignment of the sequenced ten breeds revealed 97 SNPs (Table 2) that were distributed in the following way: 24 SNPs in the 5’ UTR, 4 in the untranslated first exon, 13 SNPs in the second exon, 11 SNPs in the second intron, 3 SNPs in the third exon, 2 in the third intron, 6 SNPs in the fourth exon, 10 SNPs in the fourth intron, 9 SNPs in the fifth exon and 15 SNPs in the 3’ UTR. Considering the 31 polymorphisms detected in the coding region, 23 SNPs encode nonsynonymous mutations and 8 SNPs synonymous mutations. All the identified SNPs have been included in the submitted *SERPINA1* gene sequence (JQ436920).

**Table 2:** SNP identified in ovine *SERPINA1* gene.

**Region**	**SNP location**	**Allele variation**	**Amino acid change**	**Amino acid position**	**dbSNP ss number**
5’UTR	86	T/C	-	-	825678899
5’UTR	101	G/A	-	-	825678900
5’UTR	126	G/A	-	-	825678901
5’UTR	156	ins[TG]	-	-	825678902
5’UTR	226	T/C	-	-	825678903
5’UTR	301	G/C	-	-	825678904
5’UTR	347	A/G	-	-	825678905
5’UTR	349	G/A	-	-	825678906
5’UTR	500	T/C	-	-	825678907
5’UTR	922	G/A	-	-	825678908
5’UTR	1259	T/C	-	-	825678909
5’UTR	1275	C/T	-	-	825678910
5’UTR	1317	C/T	-	-	825678911
5’UTR	1379	G/A	-	-	825678912
5’UTR	1420	T/C	-	-	825678913
5’UTR	1441	T/C	-	-	825678914
5’UTR	1526	C/T	-	-	825678915
5’UTR	1606	A/G	-	-	825678916
5’UTR	1612	G/A	-	-	825678917
5’UTR	1659	T/C	-	-	825678918
5’UTR	1759	G/A	-	-	825678919
5’UTR	1800	C/T	-	-	825678920
5’UTR	1804	A/G	-	-	825678921
5’UTR	1870	T/C	-	-	825678922
I exon	2084	G/A	-	-	825678923
I exon	2094	T/C	-	-	825678924
I exon	2117	A/G	-	-	825678925
I exon	2144	A/T	-	-	825678926
II exon	7174	T/A	Leu/His	9	825678927
II exon	7197	T/A	Cys/Ser	17	825678928
II exon	7213	C/A	Ser/Tyr	22	825678929
II exon	7277	A/G	Ala/Ala	45	825678930
II exon	7352	C/T	Asn/Asn	68	825678931
II exon	7500	T/C	Phe/Leu	118	825678932
II exon	7567	T/C	Leu/Pro	140	825678933
II exon	7625	G/A	Leu/Leu	159	825678934
II exon	7667	G/T	Glu/Asp	174	825678935
II exon	7703	G/A	Lys/Lys	185	825678936
II exon	7711	A/G	His/Arg	188	825678937
II exon	7736	A/G	Lys/Arg	196	825678938
II exon	7741	T/A	Leu/His	198	825678939
II intron	7846	C/G	-	-	825678940
II intron	7895	A/G	-	-	825678941
II intron	8016	A/G	-	-	825678942
II intron	8044	C/A	-	-	825678943
II intron	8047	G/A	-	-	825678944
II intron	8114	C/T	-	-	825678945
II intron	8164	C/T	-	-	825678946
II intron	8219	A/G	-	-	825678947
II intron	8371	G/A	-	-	825678948
II intron	8375	C/A	-	-	825678949
II intron	8590	C/T	-	-	825678950
III exon	8701	T/C	Val/Ala	232	825678951
III exon	8745	G/A	Gly/Ser	247	825678952
III exon	8875	A/G	Asn/Ser	290	825678953
III intron	9159	G/A	-	-	825678954
III intron	9179	G/A	-	-	825678955
IV exon	9934	A/G	Glu/Glu	317	825678956
IV exon	9975	A/G	Asn/Ser	331	825678957
IV exon	9977	A/G	Arg/Gly	332	825678958
IV exon	9983	T/C	Phe/Leu	334	825678959
IV exon	9997	T/C	Ala/Ala	338	825678960
IV exon	10004	T/C	Ser/Pro	341	825678961
IV intron	10343	C/A	-	-	825678962
IV intron	10407	A/C	-	-	825678963
IV intron	10429	G/A	-	-	825678964
IV intron	10474	G/A	-	-	825678965
IV intron	10482	C/T	-	-	825678966
IV intron	10516	A/G	-	-	825678967
IV intron	10623	G/T	-	-	825678968
IV intron	10638	G/A	-	-	825678969
IV intron	10731	C/A	-	-	825678970
IV intron	10801	G/A	-	-	825678971
V exon	10834	C/G	Ala/Gly	358	825678972
V exon	10842	A/G	Thr/Ala	361	825678973
V exon	10855	A/C	Lys/Thr	365	825678974
V exon	10865	A/G	Glu/Glu	368	825678975
V exon	10901	G/A	Met/Ile	380	825678976
V exon	10920	G/A	Glu/Lys	387	825678977
V exon	10927	A/G	Asn/Ser	389	825678978
V exon	10959	A/G	Asp/Asn	400	825678979
V exon	11003	C/T	Thr/Thr	414	825678980
3’ UTR	11162	G/A	-	-	825678981
3’UTR	11506	C/T	-	-	825678982
3’UTR	11639	C/T	-	-	825678983
3’UTR	11853	G/A	-	-	825678984
3’UTR	11943	ins[T]	-	-	825678985
3’UTR	11980	G/C	-	-	825678986
3’UTR	12108	C/T	-	-	825678987
3’UTR	12219	G/A	-	-	825678988
3’UTR	12229	G/A	-	-	825678989
3’UTR	12305	G/C	-	-	825678990
3’UTR	12345	C/T	-	-	825678991
3’UTR	12479	G/A	-	-	825678992
3’UTR	12548	G/T	-	-	825678993
3’UTR	12596	G/A	-	-	825678994
3’UTR	12661	C/T	-	-	825678995

Location is based on the JQ436920 sequence; amino acid position is based on the deduced AAT protein sequence and include the 24 aa of signal peptide.

### In silico data analysis

To explain the alternative splicing events that resulted in the medium and short transcripts of ovine *SERPINA1*, we analyzed the possible influence of 54 SNPs that we identified in the gene from the second to the fifth exon, using the HSF software. The three introns had a constitutive 5’ splice donor (GT) and a constitutive 3’ splice acceptor (AG). The sequence of probable branch point sites involved in the normal splicing mechanism were cucccAc, cucugAc and cucucAc for the second, third and fourth intron respectively. None of the SNPs influenced this canonical donor, acceptor and branch point sites. The exonic and intronic mutations could impact splicing mechanism either by creating cryptic splice sites or, less frequently, by disrupting or creating exonic splicing enhancer (ESE) and exonic splicing silencer (ESS). None of 31exonic and 23 intronic SNPs resulted to have an impact on splicing of three introns of ovine *SERPINA1* gene.

To predict the possible influence of the 23 nonsynonymous aa changes on AAT function, the SIFT prediction method was used. The analysis showed that 12 aa changes could affect (scores<0.05) the protein function and 11 aa changes could be tolerate (Table 3). The aa substitutions were located in the signal peptide, in the β sheets, in the α helices, in the connection strands and in the RCL region.

**Table 3:** Result of the Sorting Intolerant From Tolerant (SIFT) analysis using 23 identified nonsynonymous aa changes in ovine AAT protein.

**Amino acid position**	**Amino acid no mutated**	**Amino acid mutated**	**SIFT score**	**Comment**	**Region of AAT**
9	L	H	0.00	affect protein function (LOW CONFIDENCE PREDICTION)	Signal peptide
17	C	S	0.00	affect protein function (LOW CONFIDENCE PREDICTION)	Signal peptide
22	S	Y	0.96	tolerated	Signal peptide
118	F	L	0.04	affect protein function	αhelix A
140	L	P	0.00	affect protein function	βSheet B
174	E	D	0.02	affect protein function	α helix F
188	H	R	0.21	tolerated	connection strand
196	K	R	0.01	affect protein function	connection strand
198	L	H	0.00	affect protein function	connection strand
232	V	A	0.00	affect protein function	β Sheet C
247	G	S	0.10	tolerated	β Sheet A
290	N	S	0.48	tolerated	connection strand
331	N	S	0.02	affect protein function	connection strand
332	R	G	0.06	tolerated	connection strand
334	P	L	0.00	affect protein function	connection strand
341	S	P	0.00	affect protein function	connection strand
358	A	G	0.01	affect protein function	β SheetB
361	T	A	0.05	tolerated	β Sheet B
365	K	T	0.59	tolerated	P16 IN RCL
380	M	I	0.19	tolerated	P1 IN RCL
387	E	K	1.00	tolerated	P7' IN RCL
389	N	S	0.02	affect protein function	P9' IN RCL
400	N	D	0.55	tolerated	connection strand

Scores less than 0.05 are predicted to be deleterious, those greater than or equal to 0.05 are predicted to be tolerated; amino acid position is based on the deduced AAT protein sequence and include the 24 aa of signal peptide

Using the default settings of NCBI, BLASTN and BLASTP search was conducted with ovine *SERPINA1* full length cDNA and deduced AAT protein as query sequences, respectively. Multi-alignments showed that the cDNA and protein were highly conserved between mammals with a 74% and 96% nucleotide similarity and 65% to 94% protein identity; specifically cDNA similarity relative to other species was: buffalo (96%), bovine (95%), swine and dog (84%), horse (82%), cat (83%), human (81%), gorilla and macaque (80%), rat (74%) and mouse (73%).

**Figure 3:**
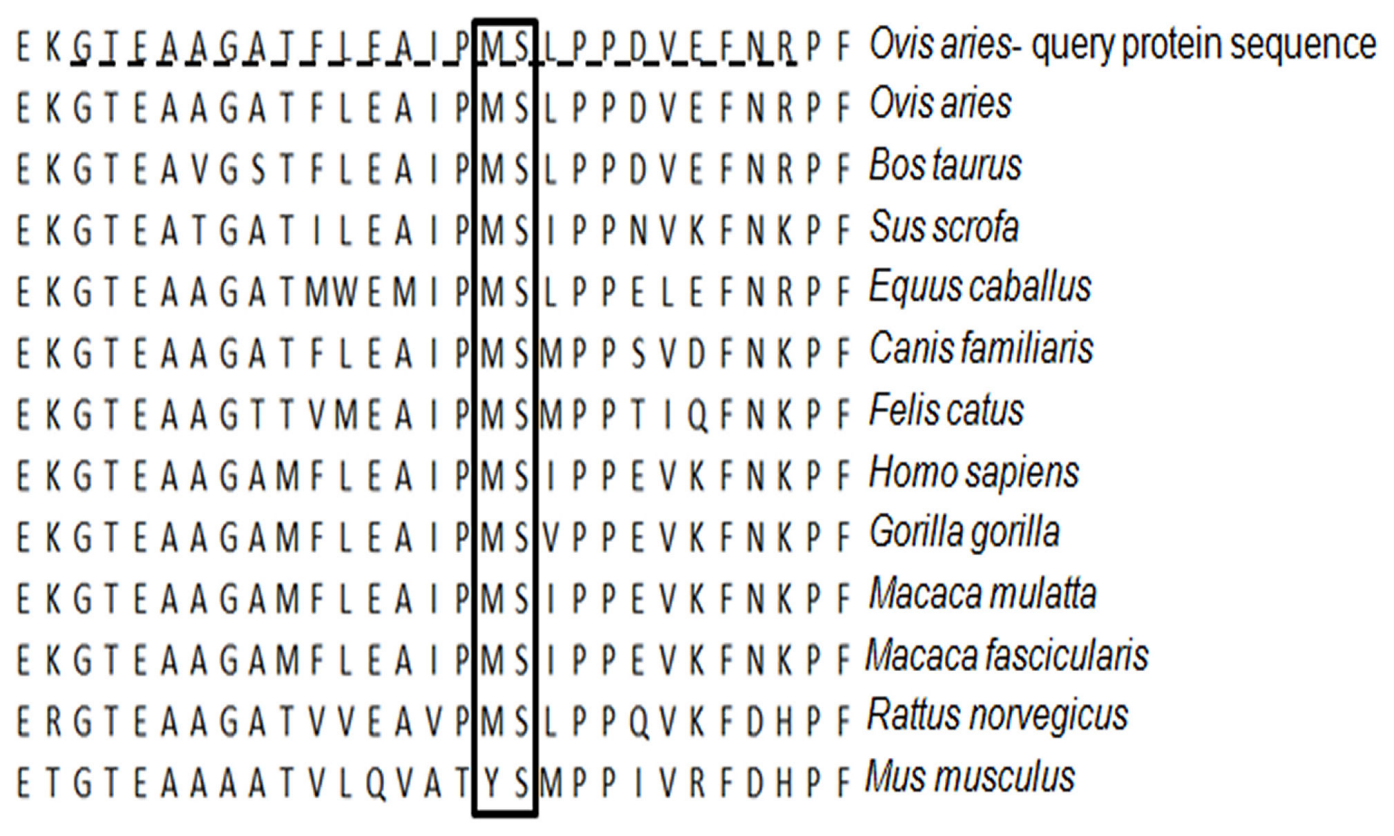
Alignment of the reactive center loop (RCL) region (25 aa) for AAT protein in different mammalian. RCL region is dotted underlined. The two amino acids important for the inhibitory AAT function are highlighted with a box.

Considering the putative protein the highest identity was with the bovine (95%) followed by swine (77%), dog and horse (75%), cat (74%), human (72%), gorilla (70%), macaque (69%), rat (65%) and mouse (62%). The amino acid sequences corresponding to the P15 to P9 positions of RCL region in different mammalian species were highly conserved (Figure 3) [3].

## Discussion

Serpins compose a large family of functionally diverse proteins. Most serpins are inhibitors of either serine or cysteine proteases involved in numerous intracellular and extracellular processes. Some serpins have non inhibitory roles such as blood pressure regulation and hormone binding [6]. Despite their different function, serpins demonstrate a highly conserved protein structure [1].

The major role of AAT is to protect tissue against proteolytic digestion by neutrophil elastase [23]. Furthermore, it has been reported that the AAT protein is present in human milk and might increase survival of other milk proteins by various mechanisms [13].

Herein, we report for the first time, 1) the isolation of three alternatively spliced ovine *SERPINA1* cDNA from milk and mammary gland; 2) the characterization of ovine *SERPINA1* gene.

Only the long transcript produces a complete AAT protein, with signal peptide, three β sheets (A, B, and C), 9 α helices (hA-hI), the region responsible for the interaction with target protease and the RCL. In silico analysis showed that both nucleotide and amino acid sequence are highly conserved in mammals. The medium transcript loses the third exon and this splicing event causes the appearance of a premature stop codon producing a shorter protein with complete elimination of the C-terminal region of protein. Because this region corresponds to the RCL region, it could be supposed that the resulting product of this transcript should not be functional. The protein produced by the shorter transcript, missing exons 2 and 3, loses the N-terminal region and part of the protein which are very important for the tridimensional folding, while it maintains the RCL region only. So we have hypothesized that the short product could have an intracellular inhibitory function given the loss of the signal peptide.

A search in the Ensembl database (http://www.ensembl.org/index.html) showed that different splicing variants are not reported in other animal species except for zebrafish (4 transcript) and human (16 transcript).

In human, Perlino et al. [24] found that *SERPINA1* gene is transcribed in macrophages from a macrophage-specific promoter different from that specific of hepatocyte cells and located about 2.0 kbp upstream. Moreover the transcription from the two *SERPINA1* promoters is mutually exclusive but in macrophages two distinct mRNAs are generated by alternative splicing. We did not find polymorphisms in the long transcript that might influence the splicing event. We analyzed only the 5’ UTR region located upstream the first exon, 2009 bp, (named for human exon A), but we did not get the sequence of the first intron, which is likely to encode further untranslated exons as shown in human hepatocyte and macrophage [24] responsible for the alternative splicing. So we have hypothesized that transcription of ovine *SERPINA1* cDNA, could be regulated from the region upstream the second exon. The hypothesis is supported by the results we obtained from *SERPINA1* gene expression analysis in 11 different tissues, where different expression profiles for the three SERPINA1 splicing variants were obtained. In *H. sapiens*, the high-throughput sequencing data have revealed that most human genes generate transcripts with different exon content also by using alternative promoters [25].

Twenty-three of the identified SNPs in the long transcript and in the gene caused nonsynonymous mutations. The polymorphism c.10901G>A (Table 2) changes methionine to isoleucine and the SIFT analysis predicted that this aa substitution is tolerated (score=0.19; Table 3). This aa substitution is tolerated because the sequence alignment produced by SIFT analysis showed that other AAT proteins (in different species), at this position, display different amino acids (non polar, uncharged polar, basic and acidic). Considering eleven mammalian species (Figure 3), methionine is always present at this position except in mouse. Moreover methionine in this position (P1 position of RCL region) has been demonstrated to be involved in the interaction of AAT with its substrates, the proteases [26,27]. Different phylogeny studies of the serpin superfamily showed the importance of the amino acid composition of the RCL region to determine the ability to bind protease and non protease ligand [1,28]. The polymorphism c.10855A>C (Table 2) caused an aa change (Lys365Thr) in P16 position of RCL region and the SIFT prediction didn’t suggest a possible influence of this mutation on AAT structure and function (score=0.59; Table 3). However the literature reports that an amino acid change at this position often converts inhibitory serpins into substrates [28], thus changing the function of the protein. Other two polymorphisms (c.10920G>A and c.10927A>G) caused aa substitutions (Glu387Lys and Asn389Ser) in two positions of RCL region (P7’ and P9’), but these aa are not crucial for conformational change of RCL region linked to substrates [3,28]. Beyond the SNPs here discussed, SIFT software predicted other aa changes likely to affect AAT function, but these were not present in positions critical for AAT inhibitory function [1,3,28]. No polymorphisms have been detected in positions P15-P 9 of RCL region. In fact ovine AAT protein displayed the consensus sequence of an inhibitory AAT [28], that provides the mobility essential for conformational changes of RCL region while interacting with the proteases.

The ovine *SERPINA1* gene exon and intron organization is similar to human and bovine. Many polymorphisms have been identified in untranslated regions (24 in the 5’ UTR and 15 in the 3’ UTR), so it would be interesting to investigate their role in controlling *SERPINA1* mRNA transcription and mRNA maturation.

Association of polymorphisms in *SERPINA1* gene with milk production traits in dairy cattle has been demonstrated [17,18]; while SNPs in *SERPINA1* gene have been reported to be associated with different human diseases, named serpinopathies [4, 12,29].

To date, the functional role of the medium and short transcripts in milk and mammary gland remains unknown. Further research should performed on the biological relevance of these transcripts and to find the molecular explanation of the alternative splicing events.

## Materials and Methods

### Collection of milk and tissue samples

Animal donors of milk and tissue samples were raised at experimental farm of CRA-ZOE (FOGGIA), research unit dealing with sheep and goat breeding for meat and milk production and extensive cattle farming. Animal management and care followed the recommendations of European Union directive 86/609/EEC. CRA-PCM (Roma) and CRA-ZOE (Foggia) are two Research Institutes settings of the Agricultural Research Council authorized by the Italian Ministry of Health to use farm animals for experimental purposes (DM 26/96-4). This research was funded by the Italian Ministry of Agriculture in the frame of GENZOOT project.

During routine morning milking ten milk samples were collected by the staff of CRA-ZOE from ewes of three different breeds (4 Sarda, 3 Gentile di Puglia and 3 Comisana) raised in the same experimental farm and traditionally managed. 50ml of milk was diluted 1:1 with PBS 1x and immediately centrifuged at 2000 g for 5 min at 4°C adding EDTA to a final concentration of 0.5 mM at pH 8.0. Fat layer was removed from the top of the supernatant with a sterile pipette tip and the skimmed milk was discarded. The cell-pellet was washed with 8 mL of buffer (0.5 mM EDTA pH 8.0 in Dulbecco’s PBS). After centrifugation, somatic cell pellet was resuspended with 1 mL TRI REAGENT (Sigma-Aldrich, Milan, Italy) reagent and stored at -80°C.

In a commercial slaughterhouse, two sheep of Sarda and Gentile di Puglia breed, were purchased in a and sacrificed following the recommendations of European Union Regulation 1099/2009. The animals were stunned by electronarcosis method and euthanized by jugular exsanguination. After slaughtering 4 g of different ovine tissue samples (spleen, *semitendinosus* and *longissimus dorsi* muscles, mammary gland, brain, cerebellum, rumen, bladder, adrenal, uterus and liver) were carefully collected and immediately submerged in 10 ml of RNA later (Sigma-Aldrich, Milan, Italy) and stored at -20°C, for RNA preservation.

### RNA and DNA extraction and quantification

RNA was extracted from somatic milk cells and tissues using the TRI REAGENT (Sigma-Aldrich, Milan, Italy) according to the manufacturer’s instructions. RNA was DNA digested by using the Rnase Free Dnase Set (Qiagen, Milan, Italy) and was then purified with the RneasyMinElute Cleanup kit (Qiagen, Milan, Italy).

### DNA was extracted following the TRI REAGENT protocol

RNA and DNA were quantified by an spectrophotometer (NanoPhotometer™ Pearl, Implen GmbH, München Germany) and quality were assessed by the spectrophotometer 260/280 ratio. For RNA only, the integrity (RIN number) was evaluated with a 2100 Bioanalyzer (Agilent Technologies, Milan, italy).

### cDNA synthesis, RT-PCR amplification and cloning, gene expression

cDNAs were synthesized from total RNA extracted from milk, mammary gland and tissue samples. Reverse transcription (RT) was performed starting from 1µg of RNA in a total volume of 20 µl containing 100 pmololigo(dT) (18-mer), 0.5 mMdNTPs, 1X RT buffer, RevertAid Premium Enzyme mix (Fermentas, M-Medical, Milan, Italy) according to the manufacturer’s instructions. The PCR amplification was done using the Dream Taq DNA polymerase (Fermentas, M-Medical, Milan, Italy) with 1µl of the first strand cDNA reaction. A touch down protocol was performed with an initial denaturation 5 min at 95°C, followed by 14 cycles of 30 sec at 94°C, 30 sec at 65°C (-0.5°C/cycle), 1 min 30 sec at 72°C; 25 cycles of 30 sec at 94°C, 30 sec at 58°C, 1 min 30 sec at 72°C; a final 5 min extension at 72°C was included. The RT-PCR amplification was performed using the primer pair that covers the full length of sheep *SERPINA1* cDNA (cDNA FWD and cDNA REV, Table 1). PCR products, obtained from milk and mammary gland samples were gel purified using Nucleospin columns (Machery-Nagel, GmbH & Co KG, Duren, Germany) and cloned in the TA cloning system (pGEM-T Easy, Promega, Milan, Italy). Four clones for each transcript were bidirectionally sequenced by using the BigDye Terminator v. 1.1 Cycle Sequencing kit and the ABI 3700 sequencer (Applied Byosystem, Life Technologies, Milan, Italy).

Only the longer transcript variant was cloned and sequenced in all 10 sheep (3 Comisana, 4 Sarda and 3 Gentile di Puglia) to detect SNPs.

For *SERPINA1* gene expression analysis in different tissues a RT-PCR amplification was performed using the same PCR protocol described above. The ATP synthase beta polypeptide (ATP5B), nuclear gene encoding mitochondrial protein, was selected as control gene. This gene is listed at http://www.primerdesign.co.uk in a list of already tested reference (house-keeping) genes inside the geNorm kits.

### PCR amplification and sequencing of the *SERPINA1* gene

cDNA FWD, cDNA REV primers (Table 1) and extracted DNA of 4 sheep (2 Comisana, 1 Gentile di Puglia e 1 Sarda) were used to amplify the complete *SERPINA1* gene. PCR protocol was: a total volume of 50 µl containing 1X Long PCR buffer with 1.5 mM MgCl_2_ (Fermentas, M-Medical, Milan, Italy), dNTPs 0.2 mM each, 1 µM forward and reverse primers, 50 ng of DNA and 0.05 U of Long PCR Enzyme mix (Fermentas, M-Medical, Milan, Italy). A two step cycling protocol was performed with an initial denaturation 3 min at 94°C, followed by 10 cycles of 30 sec 96°C, 15 sec at 68°C; 25 cycles of 10 sec at 96°C, 15 sec (+ 10 sec/cycle) at 68°C ; a 10 min final extension at 68°C was included.

The following primer pairs were used to obtain and sequence different *SERPINA1* gene fragments to cover the non-coding regions: 5’ UTR FWD and 5’ UTR REV (5’ UTR), II intron FWD and II intron REV (Ex2-Int2-Ex3), III intron FWD and III intron REV (Ex3-Int3-Ex4), IV intron FWD and cDNA REV (Ex4-Int4-Ex5), 3’ UTR FWD and 3’ UTR REV (3’ UTR) (Table 1). The primer pairs for exon-intron amplicons were designed by using our ovine *SERPINA1* cDNA sequence (GenBank accession number JQ4250369), while those for UTR regions were designed using bovine *SERPINA1* gene sequence (ID ENSEMBL: ENSBTAT00000004927).

PCR was performed in a total volume of 25 µl containing 1x DreamTaq™ Green PCR Master Mix (DreamTaq™ DNA polymerase, 1X DreamTaq™ Green buffer, dNTPs 0.2 mM each and MgCl_2_ 2 mM) (Fermentas, M-Medical, Milan, Italy), 0.4 µM forward and reverse primers and 50 ng of DNA. A touch down protocol was performed with an initial denaturation 5 min at 95°C, followed by 14 cycles of 30 sec at 94°C, 30 sec at 65°C (-0.5°C/cycle), 1 min 30 sec at 72°C; then 25 cycles of 30 sec at 94°C, 30 sec at 58°C, 1 min 30 sec at 72°C; and a 5 min final extension at 72°C.

All the PCR products were gel purified by using Nucleospin columns (Machery-Nagel, GmbH & Co KG, Duren, Germany) and were bidirectionally sequenced by using the BigDye Terminator v. 1.1 Cycle Sequencing kit and the ABI 3700 sequencer (Applied Byosystem). For the 5’ UTR and 3’ UTR amplicons, we designed internal primer pairs (Table 1) to build the whole sequence. The five fragments were sequenced in 10 sheep to detect SNPs.

### Sequence data: in silico analysis

Whole mammalian genome scanning was done to identify the homologous regions of the full length sheep SERPINA1 cDNA and gene using Basic Local Alignment Search Tool (http://www.ncbi.nlm.nih.gov/BLAST/). Sequence data were edited, translated and aligned using the free software Bioalign 4.0.6 (http://en.bio-soft.net/dna/BioLign.html). The open reading frame (ORF) of the full-length AAT cDNA was determined by ORF Finder at NCBI (www.ncbi.nlm.nih.gov/gorf/).

To identify SNPs with potential impact on splicing of SERPINA1 gene, mutant and wild sequences were analyzed with the Human Splicing Finder software (http://139.124.156.135:2300/), which includes several matrices to analyze splice sites and splicing silencers and enhancers.

To determine the potential deleterious effect of amino acid changes on protein function we used the SIFT (http://blocks.fhcrc.org/sift/SIFT.html) software. This software uses the protein sequence similarity of different species and the characteristics of amino acids (structure, polar/no polar, basic/acid) to calculate the probability of a deleterious effect of specific amino acid variants. Scores lower than 0.05 suggest a potential not tolerated amino acid substitution and a potential influence on protein function.

To search for homology of the predicted protein sequence with other species the BLASTP software was used (http://www.ncbi.nlm.nih.gov/BLAST/). We aligned the AAT protein sequences of different organisms with MEGA5 software (http://www.megasoftware.net/) [30] to examine the evolutionary conservation of RCL motifs.
